# Protective effects of green tea (*Camellia sinensis* (L.) Kuntze) against kidney diseases: a narrative review

**DOI:** 10.1590/2175-8239-JBN-2025-0349en

**Published:** 2026-09-18

**Authors:** Luis L. Ayusso, Ana Paula Girol, Luis Ayusso, Isabella Gomes, Emmanuel A. Burdmann

**Affiliations:** 1 Centro Universitário Padre Albino, Faculdade de Medicina, Divisão de Nefrologia, Catanduva, SP, Brazil.; 2 Centro Universitário Padre Albino, Departamento de Ciências Físicas e Morfológicas, Catanduva, SP, Brazil.; 3 Universidade de São Paulo, Faculdade de Medicina, Laboratório de Investigação Médica 12, São Paulo, SP, Brazil.; 4 Universidade Estadual Paulista, Instituto de Biociências, Letras e Ciências Exatas, Programa de Pós-Graduação em Biociências, São José do Rio Preto, SP, Brazil.; 5 Universidade Federal de São Paulo, Programa de Pós-Graduação em Biologia Estrutural e Funcional, São Paulo, SP, Brazil.

**Keywords:** Tea, EGCG, Oxidative Stress, Acute Kidney Injury, Renal Insufficiency, Chronic, Kidney Calculi, Nephrolithiasis, Diabetic Nephropathies

## Abstract

Kidney diseases are a global health concern, and their prevalence continues to rise. Current treatments offer limited protection for the kidneys. The protective effects of the green tea polyphenol epigallocatechin-3-gallate (EGCG) in various diseases have been widely studied. Experimental and clinical evidence indicates that the antioxidant, anti-inflammatory, and anti-apoptotic properties of EGCG demonstrate therapeutic potential for the treatment and prevention of kidney diseases. This narrative review explores the available evidence on the effects and potential protective mechanisms of EGCG across a broad range of kidney conditions, including acute kidney injury, drug-induced nephrotoxicity, kidney stones, diabetic nephropathy, chronic kidney disease, and kidney fibrosis.

## INTRODUCTION

Ethnopharmacology and ethnobotany are closely related fields. Ethnobotany examines the intricate relationships between different cultures and the use of plants, emphasizing how these plants are cultivated, utilized, and understood across different societies^1^. To effectively adopt a transdisciplinary approach in medical ethnobotany, it is essential to recognize the link between ecosystems and human communities. This connection is strengthened through the interaction between traditional and Indigenous knowledge, its sustainability, and the responsible use of medicinal plants^2^.

Ethnopharmacology is essential for understanding both modern and traditional medical practices and the biological diversity associated with this knowledge. It connects traditional knowledge of medicinal plants with scientific research, aiming to identify phytochemicals and evaluate their therapeutic potential^1,3^. Due to climate change and other human activities that damage ecosystems, there has been a gradual decline in biodiversity and Indigenous knowledge^2^. Around the world, an increasing number of people use natural products from traditional medicine to care for their health. Most individuals rely on traditional medicine and incorporate natural products as health supplements^3^. Oriental medicine (OM) still addresses many health needs of the population. In China, both modern and traditional medicine are practiced because of the extensive record of the medicinal properties of plants^1,3^. Natural products form the basis of OM, acting on multiple targets and demonstrating various pharmacological effects. For these reasons, research on them has increased with the goal of developing new medicines^3,4^. Currently, these natural compounds derived from OM and used to treat kidney diseases are widely employed worldwide. Several preclinical studies using flavonoid polyphenols from plants such as chrysin (from mushrooms and propolis), quercetin (from vegetables and fruits), morin (from mulberry), naringin (from citrus fruits like oranges and grapefruit), resveratrol (from grapes, peanuts, and berries), curcumin (from *Curcuma longa*)^5^, and catechins from green tea (*Camellia sinensis* (L.) Kuntze)^6^ have demonstrated anti-inflammatory, antioxidant, anti-apoptotic, immunomodulatory, anti-proliferative, and anti-proteinuric effects against kidney diseases. Historically, traditional Chinese medicine has been used to treat kidney disease, improving survival rates and patients’ quality of life. Nevertheless, there is a shortage of drugs for both acute and chronic kidney diseases. Traditionally, Chinese medicine has demonstrated the benefits and use of natural products in treating kidney diseases, with an emphasis on oxidative stress^5^.

### Oxidative stress, inflammation, and the kidney

Oxidative stress (OS) occurs when the antioxidant enzyme system fails or when there is an increased production of reactive oxygen species (ROS) and reactive nitrogen species (RNS)^7^. Endogenous antioxidants, such as glutathione peroxidases (GPxs), thioredoxin (Trx), catalase (CAT), and superoxide dismutase (SOD) help suppress excess ROS and maintain cellular redox balance^8^. Autophagy is a vital cellular process influenced by ROS and OS. It involves breaking down and recycling damaged organelles and proteins to sustain cellular homeostasis^8^. However, OS can cause tissue damage, inflammation, cell death, and the progression of lesions and diseases^9^. Because the kidney has a high respiratory rate and numerous mitochondria to meet its high metabolic demands, it is particularly vulnerable to oxidative damage and ROS generation^10^. Regardless of the cause, OS, autophagy activation, and mitochondrial dysfunction are interconnected in the development of kidney diseases and often occur as acute and chronic kidney injuries worsen. Many studies indicate that ROS play important roles in the development of acute kidney injury (AKI) and chronic kidney disease (CKD) across both clinical and preclinical settings^8^. The ongoing search for new therapeutic options is driven by the limited protection provided by traditional treatments. A systematic review of clinical studies on the efficacy and potential impact of natural plant bioactive compounds (LCPNs) in kidney diseases found that several LCPNs, including green tea (*Camellia sinensis*), have protective effects, mainly by activating antioxidant pathways and inhibiting pro-inflammatory signaling pathways^11^.

#### Safety considerations and potential risks linked to herbal teas

Although widely used and generally considered safe, plant-derived products, including herbal teas, carry certain risks. Evidence consistently shows that herbal preparations may cause adverse events, particularly hepatotoxicity, when used as dietary supplements or concentrated extracts^12,13^. Herb-induced liver injury is increasingly recognized in clinical practice, likely due to the rising global consumption of these products and the availability of formulations with varying quality^12^. Additionally, factors such as differences in chemical composition, extraction methods, and potential contamination can greatly affect their safety, leading to adverse events, especially in more vulnerable individuals^13,14^. It is important to note that risks are higher with concentrated extracts, which involve greater exposure to bioactive compounds at higher doses and show more variability between products, reflecting inconsistent standardization across many of them^13,14^. Therefore, a cautious approach to herbal tea consumption is advisable, particularly in populations with increased clinical vulnerability, highlighting the need for personalized assessments. Specifically, regarding green tea (*Camellia sinensis*), most adverse events are linked to concentrated catechin-rich extracts, especially when taken in high doses or on an empty stomach, while traditional green tea infusions generally present a safer profile, although rare adverse events have also been reported^13,15^.

#### Green tea (*Camellia sinensis*) – physicochemical and biological properties

*Camellia sinensis* is a species of the Theaceae family, commonly known as tea. It is a tree that can grow up to 15 meters tall, native to the forests of northeastern India and southern China. Globally, tea ranks as the second most consumed beverage, after water. The leaves of *Camellia sinensis* are used to produce this beverage. Its active chemical components have been well identified using advanced technologies over the past 40 years^16^. Among the approximately 400 chemical compounds in tea are polyphenolic compounds, or flavonoids, which can help prevent kidney disease due to their anti-inflammatory, antioxidant, and anti-apoptotic properties^11,16^.

Green tea extract (*Camellia sinensis*) is derived from leaves that undergo minimal processing to preserve phenolic compounds, especially catechins, a vital group of flavonoids^17,18^. Unlike black tea (fermented) and oolong tea (semi-fermented), green tea is classified as non-fermented because the leaves are quickly heated after harvesting, preventing catechin breakdown^17^. This is usually accomplished through methods such as steaming or light roasting, which inhibit polyphenol oxidase activity and help retain the active compounds in the beverage^17^. Afterward, the leaves are carefully dried, typically with heated air, to keep the bioactive compounds stable^17^. The plant material is then ground and extracted using hot water or hydroalcoholic solvents such as ethanol at various concentrations to maximize polyphenol recovery, especially catechins^19^. The extract may undergo additional purification steps, including filtration, vacuum concentration, and catechin enrichment. Finally, the product is dried using techniques such as spray drying or lyophilization to produce a standardized powdered extract with higher catechin content than that usually found in traditional tea infusions^15,17^. It is important to note that differences in processing and extraction methods can significantly impact the final composition, leading to variations in catechin levels and affecting dosing and routes of administration in research and clinical studies^15,18^.

Green tea includes five catechins, with only epicatechin-3-gallate (ECG) and epigallocatechin-3-gallate (EGCG) having a galloyl group. Among these, EGCG is the most abundant, making up 50–80% of the total catechin content. Several studies have demonstrated that the galloyl group in catechins is crucial for their biological properties, including antioxidant effects^20^. However, recent research has provided a theoretical basis for the anti-inflammatory mechanism of galloylated catechins (ECG and EGCG) and has shown that the presence of galloyl groups is essential for their anti-inflammatory properties^21^. EGCG is passively absorbed in the duodenum, facilitated by the alkaline pH of the intestine. This is because the small intestine lacks an active transporter to move EGCG into enterocytes and then into the bloodstream^22^. Free EGCG has limited oral bioavailability, mostly due to poor absorption and inherent instability. Its biological half-life is approximately 2–5 hours. Therefore, 24 hours after ingestion, blood levels of EGCG gradually decline to undetectable levels^22^. Although EGCG is generally well tolerated, some studies indicate that daily intake of 800 mg or more may increase blood transaminase levels^23^. Currently, EGCG is only available as a dietary supplement in the form of green tea extract and pharmaceutical products^22^. Recent advances in EGCG derivatives have shown promising properties, notably antioxidant and anti-inflammatory effects. Importantly, the most effective modified derivatives tend to have improved chemical stability and enhanced biological activity compared to unmodified EGCG. Additionally, EGCG is less stable in solution than in its solid form^22^. Considering the antioxidant properties of catechins, the distribution of hydroxyl groups is as important as their overall amount. Catechins with a pyrogallol group generally demonstrate higher antioxidant activity than those with only catechol groups. However, environmental factors also influence the antioxidant efficiency of catechins, in addition to their chemical structure^21,24^. The therapeutic potential of EGCG has been widely studied in various kidney diseases related to inflammation and OS because of its anti-inflammatory, anti-apoptotic, antioxidant, and immunoregulatory activities.

Thus, the advantages of green tea are related to the effects of EGCG, the polyphenol most thoroughly researched in medical studies. The current evidence suggests that the properties of EGCG may assist in both preventing and treating kidney diseases^11,16^.

#### EGCG – mechanism of action

**Figure 1:**
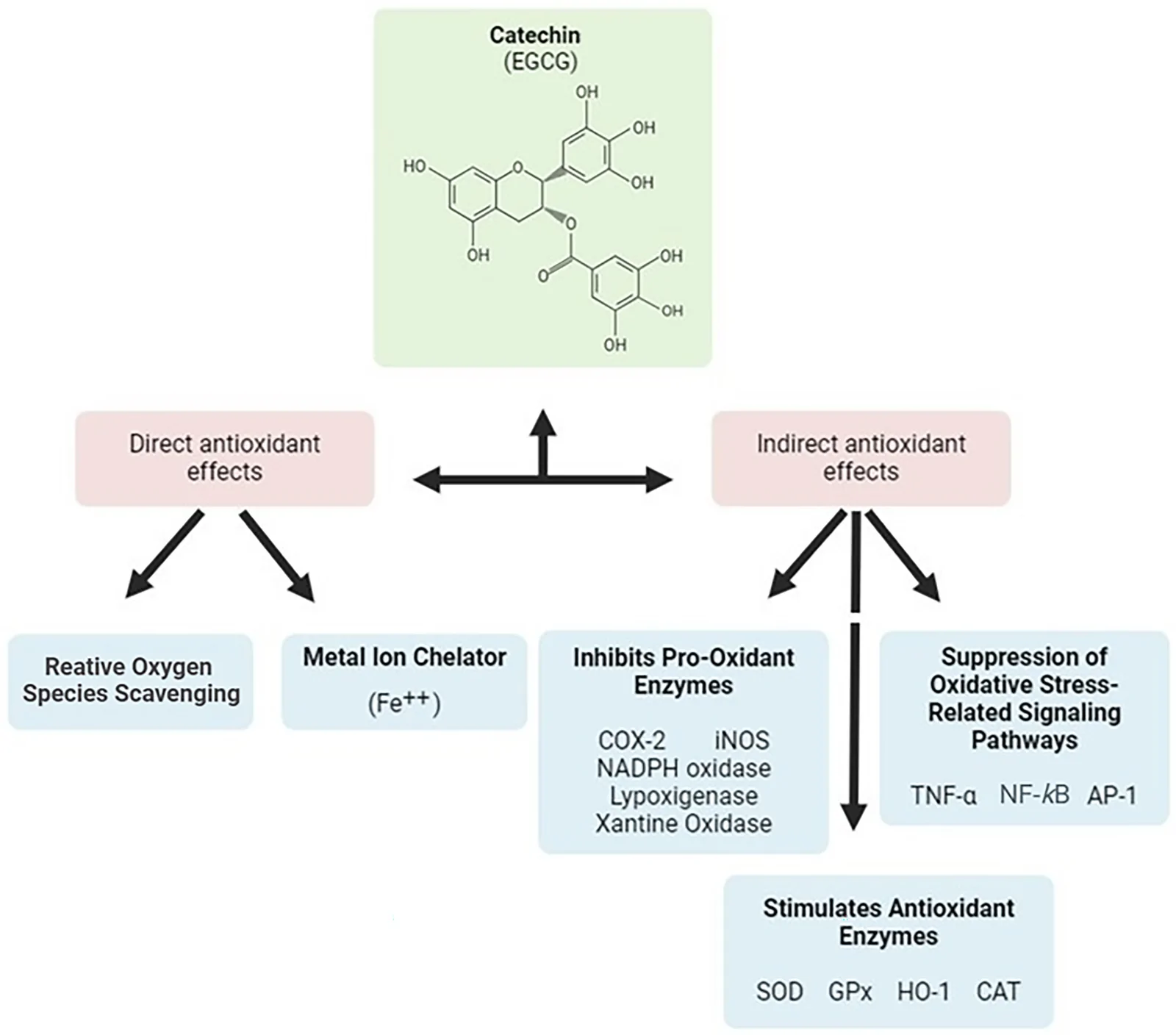
Antioxidant properties of catechins.

**Figure 2:**
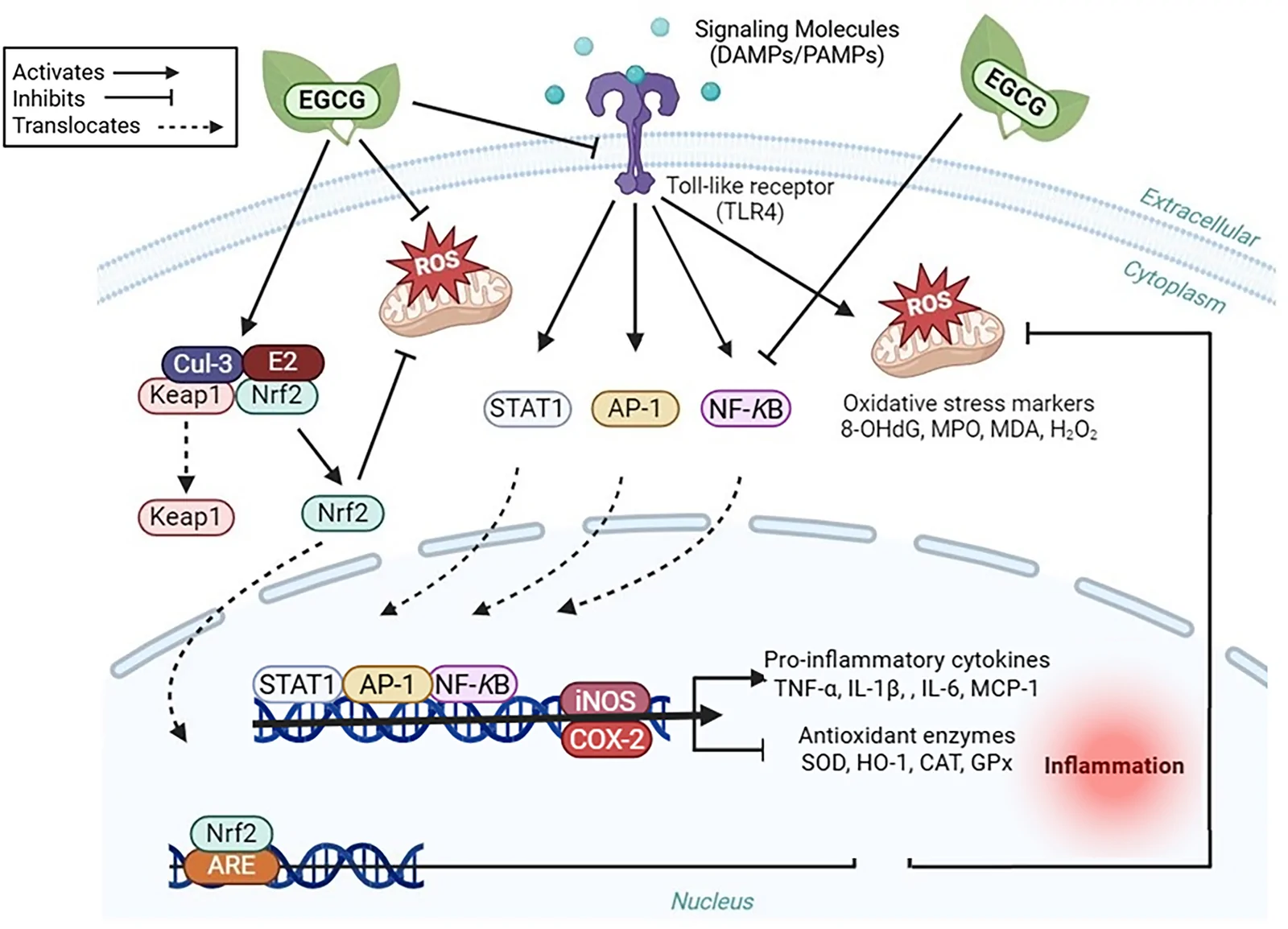
Renoprotective molecular mechanism of EGCG. EGCG specifically inhibits excessive ROS generation triggered by signaling molecules and indirectly through activation of the Nrf2/Keap1/Cul-3 signaling pathway, promoting the translocation of free Nrf2 into the nucleus, where it binds to ARE, thereby activating antioxidant enzymes and inhibiting mitochondrial ROS production. Inflammation can be inhibited by EGCG through the TLR4 and NF-κB/NLR3 signaling pathways, culminating in inhibition of the inflammatory cascade.

**Figure 3:**
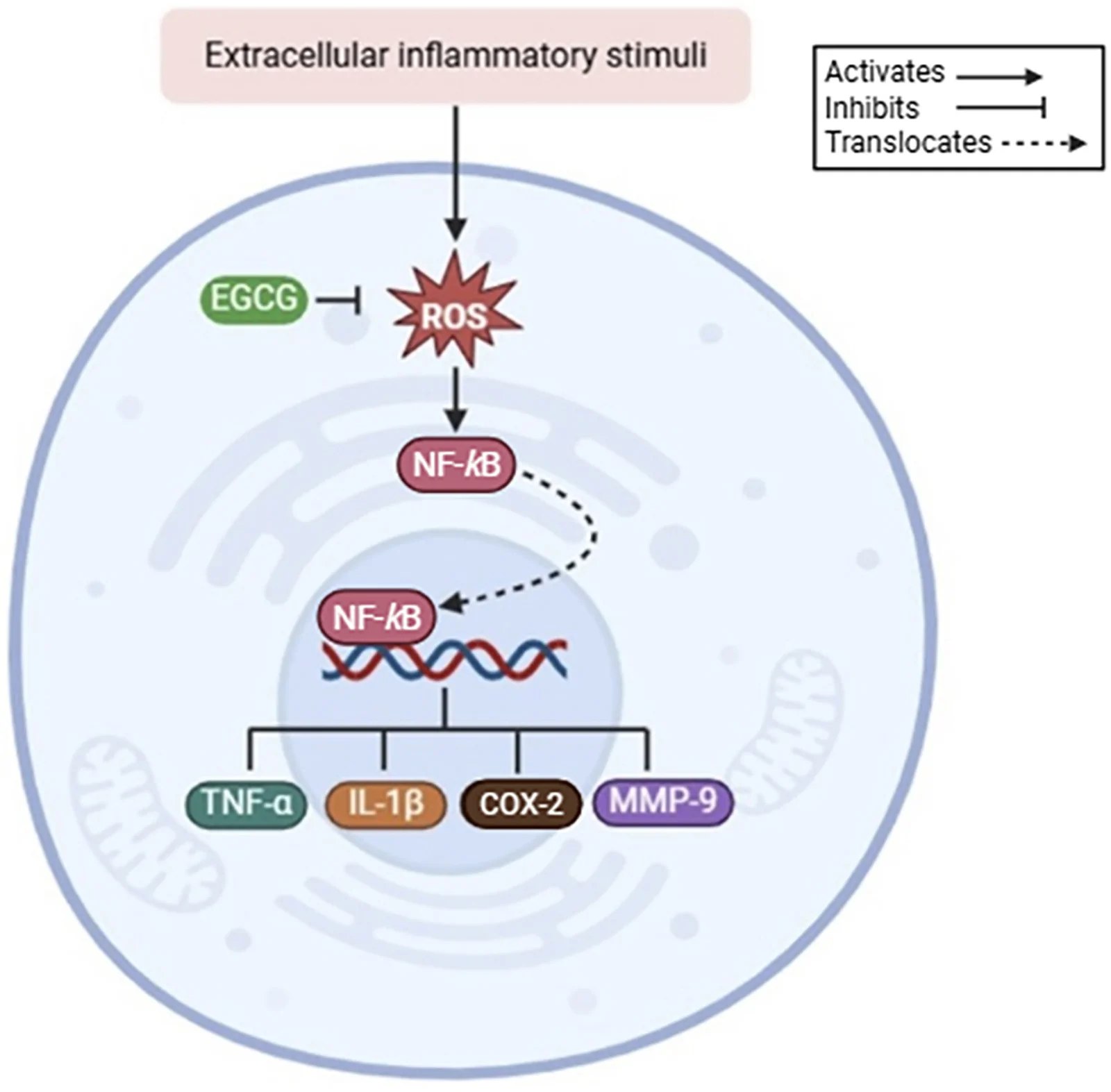
Anti-inflammatory activity of EGCG. EGCG exerts anti-inflammatory effects by reducing ROS and suppressing NF-κB signaling and its downstream effects.

The mechanism of action of green tea catechins remains unclear. According to several authors, catechins’ antioxidant properties are explained by a combination of direct and indirect processes. Direct processes involve the elimination and chelation of metal ions, such as iron, which contribute to OS. An example of an indirect process is the positive regulation of antioxidant enzymes such as SOD, CAT, and GPx, which are essential for removing reactive oxygen species (ROS). In response to OS, it is important to suppress OS-related signaling pathways, including tumor necrosis factor-alpha (TNF-α), nuclear transcription factor κB (NF-κB), and activator protein 1 (AP-1), and to inhibit pro-oxidant enzymes such as nicotinamide adenine dinucleotide phosphate (NADPH) oxidase, cyclooxygenase (COX), inducible nitric oxide synthase, xanthine oxidase, and lipoxygenase^25^ (Figure 1). Different molecular pathways, particularly those involved in the direct suppression of stimulus-induced OS, mediate the beneficial effects of green tea catechins. Other researchers support the idea that green tea’s antioxidant properties stem from preventing mitochondrial dysfunction and activating antioxidant enzymes such as heme oxygenase-1 (HO-1), SOD, GPx, and CAT through the Nrf2/Keap1/HO-1 signaling pathway. Its anti-inflammatory effects occur by inhibiting Toll-like receptor 4 (TLR4) signaling and suppressing NF-κB and NLRP3. These combined actions reduce the underlying inflammatory cascade^16^ (Figure 2). NF-κB is considered one of the major pro-inflammatory regulators of gene expression, promoting the transcription of genes encoding pro-inflammatory cytokines, such as TNF-α, IL-1β, IL-6, and IL-8, as well as inflammatory enzymes, including cyclooxygenase-2 (COX-2)^2^⁶. Green tea catechins have anti-inflammatory effects through various signaling pathways. They can provide both anti-inflammatory and antioxidant actions, with OS believed to be their main molecular target. Additionally, several researchers suggest that tea catechins may scavenge ROS, which are known to activate NF-κB signaling, leading to increased production of pro-inflammatory cytokines and enzymes such as TNF-α, IL-1β, COX-2, and matrix metalloproteinase-9 (MMP-9) (Figure 3). Therefore, the antioxidant properties of green tea may help explain its anti-inflammatory effects by preventing NF-κB activation^27^.

Abbreviations – DAMPs: damage-associated molecular patterns; TLR4: Toll-like receptor 4; STAT1: signal transducer and activator of transcription 1; NF-kB: nuclear factor kappa B; NLRP3: NOD-like receptor family pyrin domain containing 3; AP-1: activator protein 1; iNOS: inducible nitric oxide synthase; COX-2: cyclooxygenase-2; TNFα: tumor necrosis factor-alpha; IL-1β: interleukin-1β; IL-6: interleukin-6; MCP-1: monocyte chemotactic protein-1; SOD: superoxide dismutase; HO-1: heme oxygenase-1; GPx: glutathione peroxidase; Nrf2: nuclear factor erythroid 2-related factor 2; ARE: antioxidant response element; Keap1: Kelch-like ECH-associated protein 1; Cul-3: cullin-3; E2: ubiquitin-conjugating enzyme E2; H_2_O_2_: hydrogen peroxide; EGCG: epigallocatechin-3-gallate; 8-OHdG: 8-hydroxy-2´-deoxyguanosine; MDA: malondialdehyde; MPO: myeloperoxidase; ROS: reactive oxygen species.

**Figure 4:**
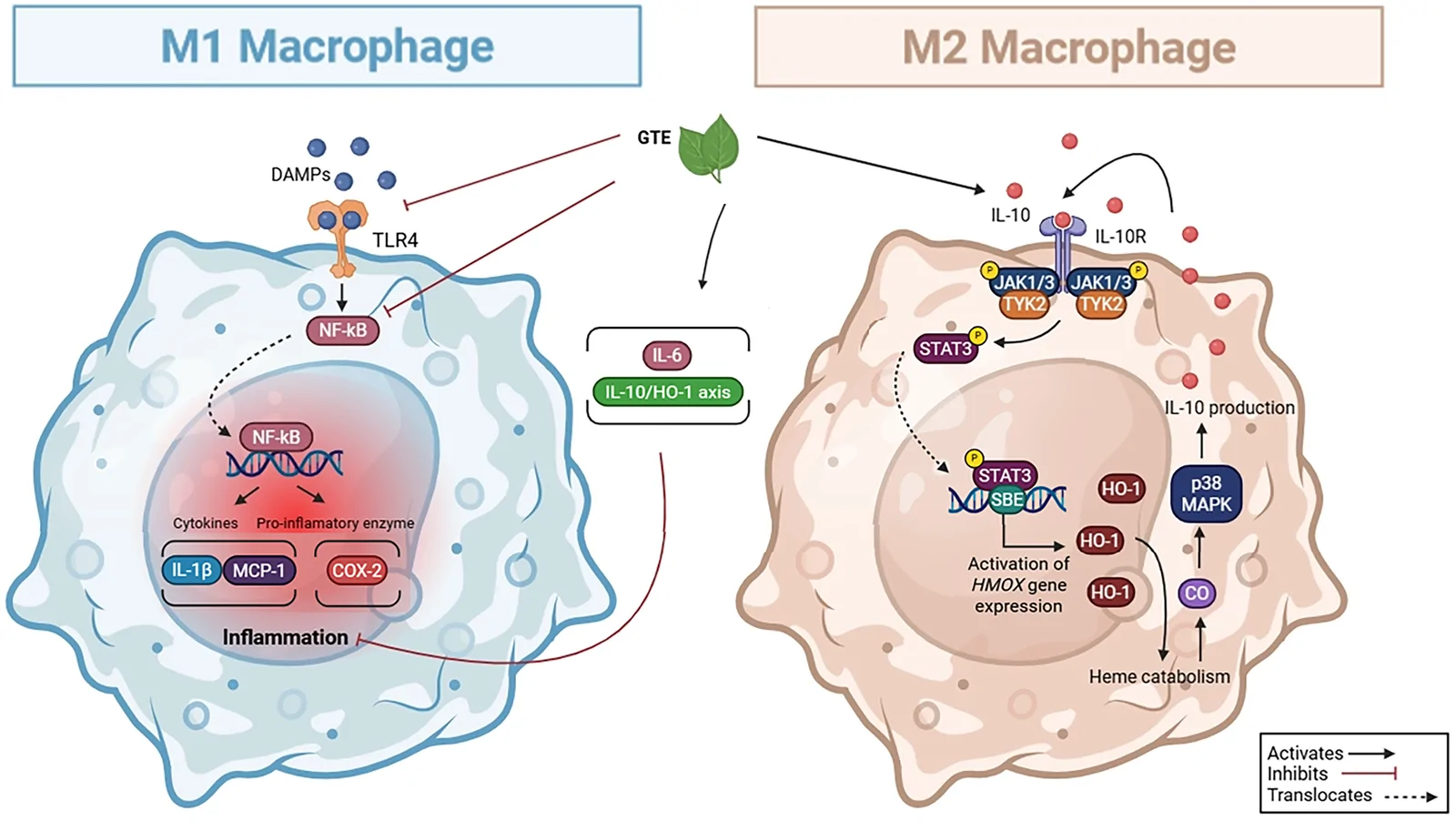
Anti-inflammatory properties of green tea extract. M1 macrophages: GTE specifically suppresses the TLR4 and NF-κB signaling pathways, leading to suppression of the underlying inflammatory cascade. IL-10/HO-1 axis in M2 macrophages. IL-10 binds to its receptor on the cell membrane and promotes the induction of JAK, which triggers the recruitment and subsequent activation of cytosolic STAT3. STAT3 is then transported to the nucleus to augment HO-1 expression. Finally, both HO-1 and CO play a role in modulating IL-10 production through p38 MAPK activation.

In contrast, Ayusso et al. recently proposed that the anti-inflammatory effects of green tea extract may result from direct inhibition of the inflammatory cascade (TLR4, NF-kB, COX-2, IL-1β, MCP-1), along with activation of the cytokine IL-6 and the IL-10/HO-1 axis in M2 macrophages, which exhibit an anti-inflammatory phenotype. This effect may occur independently of its antioxidant action^6^ (Figure 4). Our goal was to review the literature on the renoprotective effects of green tea (*Camellia sinensis*) in kidney diseases and analyze the mechanisms involved in each case.

Abbreviations – DAMPs: damage-associated molecular patterns; TLR4: Toll-like receptor 4; NF-κB: nuclear factor kappa B; COX-2: cyclooxygenase-2; IL-1β: interleukin-1β; MCP-1: monocyte chemoattractant protein-1; GTE: green tea extract; IL-10: interleukin-10; HO-1: heme oxygenase-1; IL-10R: interleukin-10 receptor; STAT3: signal transducer and activator of transcription 3; SBE: STAT-binding element; CO: carbon monoxide; MAPK: mitogen-activated protein kinase; JAK1/3: Janus kinase; and TYK2: tyrosine kinase 2.

## METHODS

A narrative review was performed through a structured search of the PubMed/MEDLINE database to identify studies published between January 2014 and December 2024 that examined the effects of green tea and its main bioactive compound, EGCG, on kidney diseases. The search was conducted in December 2024, with that date set as the cutoff for the inclusion of eligible studies. A total of 2,190 records were identified, with no duplicates found during the selection process. After screening titles and abstracts, 2,000 articles were excluded for being irrelevant to the topic. The remaining 190 studies were reviewed in full, and 127 were excluded for not meeting the eligibility criteria. Ultimately, 63 studies were included in this review.

For article identification, a combination of controlled descriptors and free-text terms related to green tea, its primary bioactive compounds, and various renal conditions was used. These included terms such as green tea, *Camellia sinensis*, epigallocatechin gallate, EGCG, oxidative stress, kidney disease, renal disease, acute kidney injury, chronic kidney disease, nephrolithiasis, and diabetic nephropathy. These terms were combined using the Boolean operators AND and OR to enhance search sensitivity. An example search strategy was: (“green tea” OR “*Camellia sinensis*” OR EGCG OR “epigallocatechin gallate”) AND (“kidney disease” OR “renal disease” OR “acute kidney injury” OR “chronic kidney disease” OR “nephrolithiasis” OR “diabetic nephropathy”) AND (“oxidative stress”).

### Inclusion criteria

Original experimental and clinical studies that examined the renoprotective effects of green tea or its primary bioactive polyphenol, especially EGCG, on various kidney diseases were included. Eligible articles were published between January 2014 and December 2024, written in English, and indexed in PubMed/MEDLINE.

### Exclusion criteria

Studies that did not specifically examine the effects of green tea or its bioactive compounds on kidney diseases were excluded, along with review articles, letters to the editor, editorials, conference abstracts without full-text access, and publications lacking original experimental or clinical data relevant to the purpose of the review.

The process of study selection is summarized in the flowchart (Figure S1).

## RESULTS

### Protective effect of green tea in kidney diseases

#### Chronic kidney disease (CKD)

CKD is increasingly prevalent worldwide and is linked to diabetes and hypertension. Multiple molecular signaling pathways are involved in the initiation and progression of kidney injury. The renin–angiotensin–aldosterone system and the stimulation of profibrotic growth factors, such as transforming growth factor β (TGF-β) and connective tissue growth factor, are part of this process. TGF-β enhances ROS production, suppresses the antioxidant system, and induces OS or cellular redox imbalance. This cascade leads to extracellular matrix (ECM) accumulation, kidney fibrosis, epithelial dysfunction, and pro-inflammatory responses. These pathways not only overlap but also interact, altering their biological roles and promoting the progression of kidney fibrosis and CKD^11^. A recent literature review focused on the primary sources of ROS, endogenous antioxidant defenses, and redox-sensitive signaling pathways involved in inflammation regulation and fibrosis in CKD. The authors noted that the antioxidant enzyme system (e.g., SOD, CAT, GPx) plays a crucial role in mitigating ROS-induced renal injury. In contrast, NADPH oxidase and mitochondria are the main sources of ROS in the kidney^28^. Therapeutic options to prevent these pathological progressions are limited. Drugs that block the renin–angiotensin–aldosterone system only partially reduce proteinuria, improve kidney function, and delay progression to end-stage kidney disease^29^. The protective effects of EGCG on the kidney have been extensively studied in preclinical models of kidney fibrosis and CKD.

EGCG acts as an antioxidant by activating the Nrf2–HO-1 pathway, leading to the production of antioxidant enzymes (SOD, CAT, GPx) and helping maintain mitochondrial function. Its antifibrotic effects are achieved by suppressing the TGF-β/Smad pathway and epithelial–mesenchymal transition. Additionally, EGCG exhibits anti-apoptotic properties by inhibiting p38 MAPK signaling. Its anti-inflammatory effect stems from blocking the NF-κB pathway, which regulates the expression of pro-inflammatory chemokines and cytokines such as TNF-α, IL-1β, and IL-6, as well as the activity of the enzyme myeloperoxidase^28,30^. Several studies have shown that treatment with EGCG reduces serum creatinine levels in preclinical models of obstructive nephropathy^16^.

#### Nephrolithiasis

Recently, it was confirmed that kidney epithelial cell injury related to calcium oxalate (CaOx) stone formation is mediated by OS, which is produced by mitochondria or NADPH oxidase^31^. The roles of OS and endoplasmic reticulum stress in calcium nephrolithiasis suggest that the connection between oxalate toxicity and endoplasmic reticulum stress may contribute to stone formation. However, the interaction between reactive oxygen species (ROS) and endoplasmic reticulum stress remains debated. Research shows that CaOx crystal formation and OS are key factors in the development of nephrolithiasis^32^. Administering green tea polyphenols inhibits free radical production induced by CaOx monohydrate (COM) in kidney tubular epithelial cells, promotes the formation of CaOx dihydrate (COD), and reduces the expression of α-enolase (a receptor for crystalline COM) on cell surfaces, as well as decreasing oxaluria. The shape of crystals and kidney cell aggregates may influence CaOx attachment to kidney tubular cells, which is critical for stone development. A signaling pathway triggered by green tea catechins that combats OS related to kidney stone formation involves increasing the levels of antioxidant enzymes, such as SOD, GPx, and CAT^33,34^. Few clinical trials have explored the relationship between green tea and nephrolithiasis. One large cohort study in the United Kingdom, involving 439,072 participants followed for an average of over six years, found that individuals who drank five cups of green tea daily had a significantly lower risk of kidney stones than those who did not drink tea regularly^34^. Recently, a systematic review evaluated the available evidence on the relationship between tea consumption and nephrolithiasis risk^35^. The review included 13 non-randomized studies with varying designs and lacked standardized tea preparation methods, indicating publication bias. Due to differences in procedures and data, a meta-analysis was not possible. The review suggested that tea might help prevent kidney stones because of its antioxidant effects, hydration benefits, and caffeine content^35^. A recent prospective cohort study in China, involving 502,621 adults followed for a median of over 11 years, found that drinking three cups of tea daily, consuming 30 g of alcohol, and eating fruit four times a week were associated with a lower incidence of kidney stones^36^. Higher tea intake, along with fruit consumption and moderate alcohol use, was independently associated with lower rates of nephrolithiasis^36^. Nutritional factors significantly influence the development of urolithiasis. A narrative review provided a comprehensive, updated overview of the effects of nutrition and diet on nephrolithiasis. Large cohort studies supporting the benefits of coffee and tea intake suggest that tea’s positive effects are mainly due to its diuretic properties, related to caffeine, which may help reduce hypercalciuria. The antioxidant properties of polyphenols could also help explain tea’s protective effects. However, these cohort studies had one limitation: they did not distinguish between black and green tea, which differ in oxalate levels depending on factors such as origin, quality, harvest time, and preparation methods. Therefore, the exact mechanisms by which black and green tea inhibit stone formation remain uncertain^37^.

#### Diabetic nephropathy

Diabetes mellitus can lead to both macrovascular and microvascular complications, including diabetic nephropathy (DN), a microvascular disease that is highly feared in clinical practice. DN involves impaired kidney filtration and proteinuria. Both experimental and human studies have indicated that tea offers protective effects against diabetes mellitus and helps manage its related complications. Evidence suggests that tea may influence the mechanisms underlying DN through its antioxidant, anti-inflammatory^38,39^, anti-apoptotic, and anti-fibrotic properties^16,40^, thereby reducing proteinuria and protecting kidney function^16,38,40^. A randomized, double-blind, placebo-controlled trial with 42 patients with DN demonstrated that green tea extract (polyphenols), when used with maximum-dose angiotensin II receptor blockers (ARBs) and/or angiotensin-converting enzyme inhibitors, was safe and effective, leading to decreased proteinuria and less podocyte apoptosis via the Wnt pathway^41^. Multinational epidemiological studies in the USA, China, Japan, and South Korea involving diverse racial, sex, and age groups have shown that tea consumption can lower the risk of developing diabetes and its complications, including nephropathy. Most participants consumed black, oolong, or green tea. The antioxidant EGCG can reduce OS and inflammation by inducing the Nrf2 signaling and inhibiting NF-κB pathways. Due to variability in tea dosages and the effects observed in preclinical and clinical research, uncertainty remains about whether doses effective in preclinical studies can produce similar benefits in humans^38^. Hyperglycemia can trigger OS, the formation of advanced glycation end-products (AGEs), activation of the polyol pathway, and abnormal activation of protein kinase C (PKC), all of which contribute to DN. Elevated blood glucose levels increase α-diacylglycerol (α-DAG) synthesis, which abnormally activates PKC, leading to DN and other vascular complications. Upregulating α-diacylglycerol kinase (α-DGK) inhibits PKC activation and decreases α-DAG levels. Preclinical studies in streptozotocin-induced type I diabetic mice have shown that EGCG significantly reduces albuminuria in DN by activating the α-DGK signaling pathway. These studies also found that EGCG activates α-DGK via the 67 kDa laminin receptor (67LR), known as the EGCG receptor. The findings suggest that EGCG triggers α-DGK activation through 67LR and c-Src, with α-DGK associating with 67LR in podocyte membranes to inhibit PKC. Upon activation by EGCG, complexes of α-DGK, c-Src, and 67LR appear to form. Researchers concluded that activating the α-DGK pathway could be a promising new therapeutic target for DN^42^. Strategies to slow DN progression include lifestyle modifications, dietary guidance, regular physical activity, and the use of renoprotective drugs like ARBs and ACE inhibitors. The main biochemical pathway involved in disease progression is mediated by advanced glycation end-products (AGEs), which bind to their receptor (RAGE) and modulate intracellular signaling that may promote or worsen kidney injury. Soluble RAGE (sRAGE), a RAGE variant, interacts with AGEs and may help inhibit this signaling. One proposed mechanism for DN development involves elevated AGEs binding to RAGE, causing damage to nephron cells. Green tea has potentially been shown to increase sRAGE expression; however, it remains unclear whether this effect improves renal function. A randomized, double-blind, placebo-controlled clinical trial conducted in Guadalajara, Mexico, examined the impact of green tea extract on sRAGE levels and kidney damage in people with type 2 diabetes^43^. The study involved 39 men and women aged 35 to 65, all on oral hypoglycemic agents and/or insulin, with chronic kidney disease (CKD) stages 2 or 3a according to KDIGO guidelines, and HbA1c levels between 9 and 12%. Participants received 400 mg of green tea twice daily or a placebo for three months. The researchers observed that green tea extract significantly increased serum sRAGE levels and glomerular filtration rate while lowering fasting blood glucose and triglyceride levels^43^.

#### Acute kidney injury

Acute kidney injury (AKI) refers to a sudden loss of kidney function. Serum creatinine and urine output are key indicators for diagnosing AKI, although their limitations are well recognized^44^. AKI remains a major global health issue, affecting approximately 13.3 million people worldwide each year^45^. During the COVID-19 pandemic, AKI was common and associated with higher rates of morbidity and mortality. Multiple studies have shown that EGCG can act as a protective or therapeutic agent in AKI. Its protective effects may include antioxidant, anti-inflammatory, and anti-apoptotic activities. The main signaling pathways involved are activation of the Nrf2–HO-1 pathway and suppression of NF-κB via MAPK^16,46^. Studies in rats pretreated or treated with catechins demonstrated significant protection or reduction of kidney damage, including glomerular and tubular injury, in models of AKI caused by gentamicin, contrast agents, rhabdomyolysis, cisplatin, glycerol, gamma radiation, heat stress, obstruction, ischemia/reperfusion, cardiopulmonary bypass, iron overload, cyclosporine, tacrolimus, and doxorubicin^6,16,42,47,48,49,50,51,52,53^. Recent research indicates that catechins also have antiviral effects and can prevent COVID-19-related AKI^45,54^. Catechins have demonstrated antiviral properties against coronaviruses and other RNA viruses, and they can inhibit endocytosis, replication, and transcription through mechanisms that are not yet fully understood. It is well established that EGCG interacts with the ACE2 receptor on host cell membranes, as well as with RNA-dependent RNA polymerase (RdRp) and the main protease (Mpro), thereby inhibiting SARS-CoV-2 replication. The ACE2 receptor serves as the binding site for the spike protein of SARS-CoV-2, specifically via the receptor-binding domain (RBD) within the viral S1 subunit^54^. The interaction between the SARS-CoV-2 spike protein and the ACE2 receptor is mediated by the RBD, located in the S1 subunit. This facilitates recognition of ACE2 by the RBD and the formation of the RBD–ACE2 complex. Once this complex forms, SARS-CoV-2 can undergo endocytosis, initiating replication. *In vitro* studies have shown that EGCG binds to the SARS-CoV-2 spike protein, blocking its interaction with ACE2 on host cells and preventing viral entry^45^. Additionally, EGCG inhibits early stages of viral replication by targeting Mpro and RdRp^55^. These findings support the potential of tea catechins as a renoprotective therapy for COVID-19-related AKI, owing to their anti-SARS-CoV-2, antioxidant, and anti-inflammatory properties^45^.

Table 1 summarizes the main experimental and clinical studies included in this review, highlighting the routes of administration, doses used, intervention durations, key renal outcomes assessed, and the safety profile of green tea and its catechins, especially EGCG.

**Table 1:** Summary of studies on green tea and EGCG in renal diseases.

Study (author, year)	Condition	Study design	Administration	Dose	Duration	Main outcome	Safety
Li et al., 2021	Nephrolithiasis	Experimental	Polyphenols (gavage)	30–300 mg/kg/day	4 Weeks	↓ crystal formation	Well tolerated
Littlejohns et al., 2019	Nephrolithiasis	Cohort	Tea infusion	~5 cups/day	> 6 yrs.	↓ stone risk	No significant adverse effects
Wang et al., 2021	Nephrolithiasis	Cohort	Tea infusion	≥ 3 cups/day	> 11 yrs.	↓ incidence of nephrolithiasis	No significant adverse effects
Borges et al., 2016	Diabetic nephropathy	Randomized clinical trial (RCT)	Extract	400 mg twice daily	12 weeks	↓ proteinuria	Well tolerated
Hayashi et al., 2020	Diabetic nephropathy	Experimental	EGCG (STZ model)	0.1% in diet (w/w)	8 weeks	↓ albuminuria	Well tolerated
Barocio et al., 2021	Diabetic nephropathy	Randomized clinical trial (RCT)	Extract	400 mg twice daily	3 months	↑ kidney function and metabolic parameters	Well tolerated
Multiple studies	AKI	Experimental	EGCG/extract	Variable	Variable	Tubular protection	Potential hepatotoxicity at high doses

## DISCUSSION AND CONCLUSIONS

**Figure 5:**
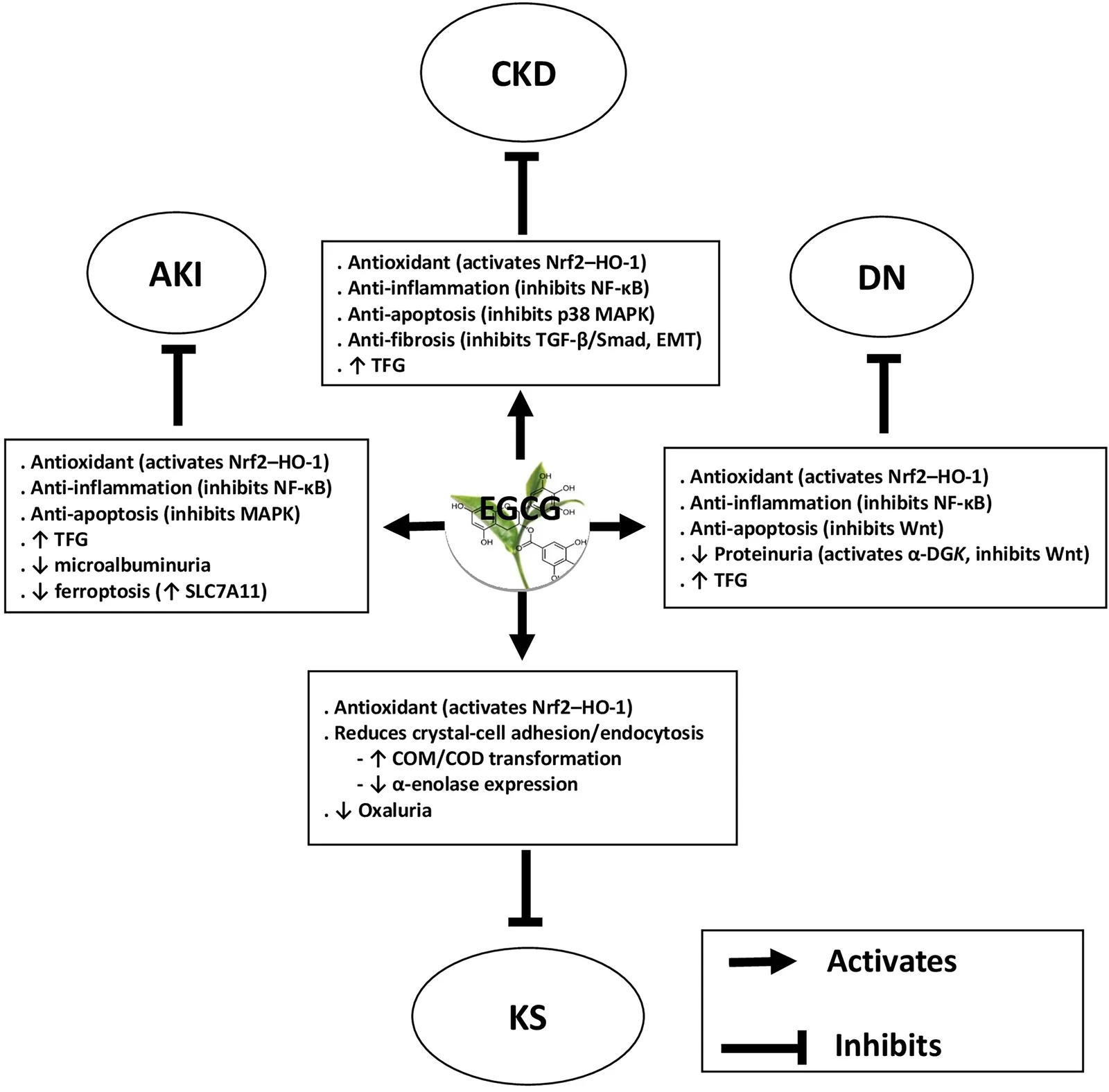
Renoprotective effects of EGCG in various kidney diseases. EGCG protects against kidney diseases through multiple pathways, primarily via its antioxidant and anti-inflammatory activities.

Herbal medicines frequently face challenges, including a lack of standardization, variability in composition, and poor-quality control, which can impact their safety and clinical reliability. These problems are worsened by differences in regulatory frameworks and pharmacovigilance systems across countries, especially in low and middle-income regions, including Brazil^56^. The safety of green tea catechins, especially EGCG, should be considered when evaluating their therapeutic potential in kidney diseases. Evidence from the European Food Safety Authority shows that high doses, particularly ≥ 800 mg/day in concentrated extracts, may cause hepatic abnormalities^15,23^, while lower doses have not consistently been linked to liver toxicity. Factors such as bolus dosing, fasting status, and formulation details may also influence its safety profile^13,15^. Nevertheless, data from the United States Pharmacopeia suggest that severe hepatotoxicity associated with green tea extracts is rare, despite their widespread use worldwide^13^. When adverse events occur, they mainly appear to be related to individual factors, including genetic predisposition, idiosyncratic reactions, use of hepatotoxic drugs, pre-existing liver disease, and formulation and use-related factors^13,15^. Clinical studies show that EGCG has good tolerability, especially at moderate doses^41,43^, with no consistent signs of toxicity, although safety may depend on dose and formulation^15^. Consistently, clinical trials (Borges et al.^41^ and Barocio-Pantoja et al.^43^) using doses of 800 mg/day reported no adverse effects, supporting the favorable safety and tolerability profile of the compound (Table 1). The management of chronic kidney disease, especially diabetic kidney disease, has advanced with the inclusion of renoprotective therapies that slow disease progression, reduce cardiovascular risk, and lower mortality. These include SGLT2 inhibitors (dapagliflozin, empagliflozin), mineralocorticoid receptor antagonists (finerenone), and GLP-1 receptor agonists (liraglutide)^57,58,59,60,61^. However, a significant residual risk of progression remains despite renin–angiotensin system blockade^62^. Given the increasing global burden of kidney diseases ^63^, new therapeutic approaches are still needed. The synthesis of the included studies (Figure 5 and Table 1) indicates promising renoprotective effects of EGCG, though significant methodological heterogeneity and variability in formulations limit its clinical application. These effects seem to involve modulation of OS and inflammation, especially through the Nrf2–Keap1 and NF-κB pathways. We conclude that EGCG could be a useful adjunct to established therapies, though well-designed clinical trials are necessary to determine its role in clinical practice.

Abbreviations – AKI: acute kidney injury; DN: diabetic nephropathy; CKD: chronic kidney disease; KS: kidney stones; Nrf2: nuclear factor erythroid 2-related factor 2; HO-1: heme oxygenase-1; EGCG: epigallocatechin-3-gallate; NF-κB: nuclear factor kappa-light-chain-enhancer of activated B cells; MAPK: mitogen-activated protein kinase; GFR: glomerular filtration rate; TGF-β: transforming growth factor-β; EMT: epithelial–mesenchymal transition; α-DGK: α-diacylglycerol kinase; COM/COD: transformation of calcium oxalate monohydrate (COM) crystals into calcium oxalate dihydrate (COD) crystals.

## Data Availability

The datasets generated and/or analyzed during this study are available from the corresponding author upon reasonable request.

## SUPPLEMENTARY MATERIAL

The following online material is available for this article:

Figure S1 – Study selection flow diagram.
