# Supplementary material for: Protective effects of green tea (Camellia sinensis (L.) Kuntze) against kidney diseases: a narrative review
Source: J Bras Nefrol. 2026 Sep 18;48(4):e20250349. doi: 10.1590/2175-8239-JBN-2025-0349en (PMC13600745; doi:10.1590/2175-8239-JBN-2025-0349en)
Supplement: Supplementary file 2 [file 2175-8239-jbn-48-4-e20250349-suppl2.pdf]

Braz. J. Nephrol.
https://doi.org/10.1590/2175-8239-JBN-2025-0349pt

Material Suplementar para "Efeitos protetores do chá verde (*Camellia sinensis* (L.) Kuntze) sobre as doenças renais: revisão narrativa"

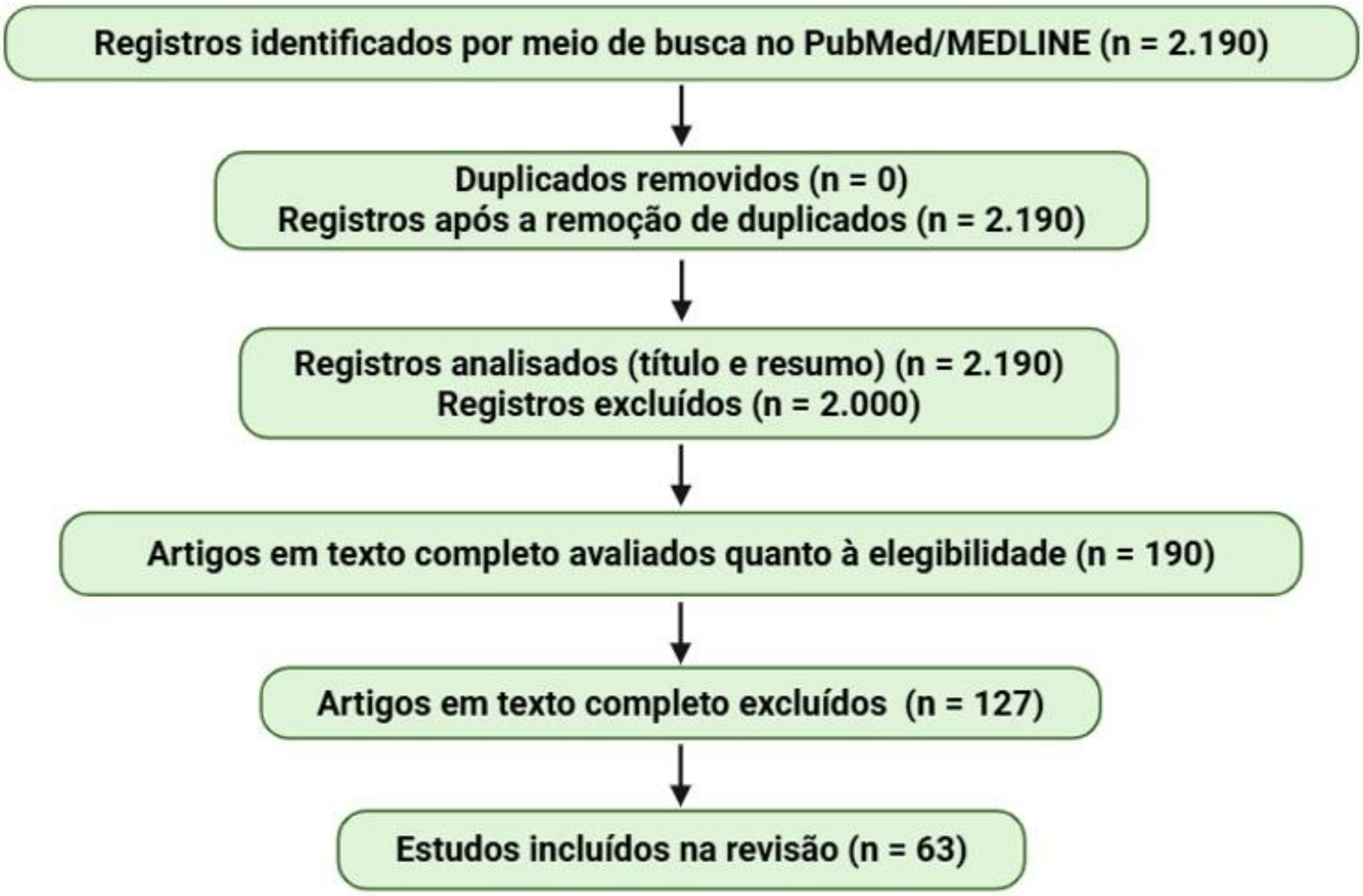

Figura S1. Fluxograma de seleção dos estudos.
