# Supplementary material for: Protective effects of green tea (Camellia sinensis (L.) Kuntze) against kidney diseases: a narrative review
Source: J Bras Nefrol. 2026 Sep 18;48(4):e20250349. doi: 10.1590/2175-8239-JBN-2025-0349en (PMC13600745; doi:10.1590/2175-8239-JBN-2025-0349en)
Supplement: Translation

# Translation: Efeitos protetores do chá verde (*Camellia sinensis* (L.) Kuntze) sobre as doenças renais: revisão narrativa

**DOI:** 10.1590/2175-8239-JBN-2025-0349pt

**Authors:** Luis L. Ayusso, Ana Paula Girol, Luis Ayusso, Isabella Gomes, Emmanuel A. Burdmann

**Affiliations:** 1 Centro Universitário Padre Albino, Faculdade de Medicina, Divisão de Nefrologia, Catanduva, SP, Brasil.; 2 Centro Universitário Padre Albino, Departamento de Ciências Físicas e Morfológicas, Catanduva, SP, Brasil.; 3 Universidade de São Paulo, Faculdade de Medicina, Laboratório de Investigação Médica 12, São Paulo, SP, Brasil.; 4 Universidade Estadual Paulista, Instituto de Biociências, Letras e Ciências Exatas, Programa de Pós-Graduação em Biociências, São José do Rio Preto, SP, Brasil.; 5 Universidade Federal de São Paulo, Programa de Pós-Graduação em Biologia Estrutural e Funcional, São Paulo, SP, Brasil.

**Keywords:** Chá, EGCG, Estresse Oxidativo, Injúria Renal Aguda, Insuficiência Renal Crônica, Cálculos Renais, Nefrolitíase, Nefropatias Diabéticas.

## Abstract

As doenças renais constituem um importante problema de saúde global, cuja prevalência continua a aumentar. As terapias atualmente disponíveis oferecem proteção limitada aos rins. Os efeitos protetores do polifenol do chá verde, a epigalocatequina-3-galato (EGCG), em diversas doenças têm sido amplamente investigados. Evidências experimentais e clínicas indicam que as propriedades antioxidantes, anti-inflamatórias e antiapoptóticas do EGCG apresentam potencial para o tratamento e a prevenção de doenças renais. Esta revisão narrativa explora as evidências disponíveis sobre os efeitos e os potenciais mecanismos protetores do EGCG em um amplo espectro de condições renais, incluindo injúria renal aguda, nefrotoxicidade induzida por fármacos, nefrolitíase, nefropatia diabética, doença renal crônica e fibrose renal.

## INTRODUÇÃO

A etnofarmacologia e a etnobotânica são campos estreitamente relacionados. A etnobotânica examina as complexas relações entre diferentes culturas e o uso de plantas, enfatizando como essas são cultivadas, utilizadas e compreendidas em diversas sociedades^1^. Para adotar de forma eficaz uma abordagem transdisciplinar na etnobotânica médica, é essencial reconhecer a ligação entre os ecossistemas e as comunidades humanas. Essa conexão é fortalecida por meio da interação entre o conhecimento tradicional e indígena, sua sustentabilidade e o uso responsável das plantas medicinais^2^.

A etnofarmacologia é essencial para a compreensão tanto das práticas médicas modernas quanto das tradicionais e da diversidade biológica associada a esse conhecimento. Ela conecta o saber tradicional sobre plantas medicinais à pesquisa científica, com o objetivo de identificar fitoquímicos e avaliar seu potencial terapêutico^1,3^. Devido às mudanças climáticas e a outras atividades humanas que degradam os ecossistemas, tem-se observado um declínio gradual da biodiversidade e do conhecimento indígena^2^. Em todo o mundo, um número crescente de pessoas recorre à medicina tradicional para cuidar da saúde, incorporando produtos naturais como suplementos terapêuticos^3^. A medicina oriental (MO) ainda atende a muitas necessidades de saúde da população. Na China, tanto a medicina moderna quanto a tradicional são praticadas devido ao extenso registro das propriedades medicinais das plantas^1,3^. Os produtos naturais constituem a base da MO, atuando em múltiplos alvos e demonstrando diversos efeitos farmacológicos. Por essas razões, a pesquisa nessa área tem aumentado com o objetivo de desenvolver novos medicamentos^3,4^. Atualmente, compostos naturais derivados da MO são amplamente empregados no tratamento de doenças renais em todo o mundo. Diversos estudos pré-clínicos utilizando polifenóis flavonoides de plantas, como crisina (de cogumelos e própolis), quercetina (de vegetais e frutas), morina (da amoreira), naringina (de frutas cítricas, como laranja e *grapefruit*), resveratrol (de uvas, amendoim e frutas vermelhas), curcumina (de *Curcuma longa*)^5^ e as catequinas do chá verde (*Camellia sinensis* (L.) Kuntze)^6^ demonstraram efeitos anti-inflamatórios, antioxidantes, antiapoptóticos, imunomoduladores, antiproliferativos e antiproteinúricos em doenças renais. Historicamente, a medicina tradicional chinesa tem sido utilizada no tratamento de doenças renais, melhorando as taxas de sobrevida e a qualidade de vida dos pacientes. Ainda assim, há escassez de fármacos tanto para doenças renais agudas quanto para as crônicas. Tradicionalmente, a medicina chinesa tem demonstrado o uso e os benefícios de produtos naturais no tratamento dessas doenças, com ênfase no estresse oxidativo^5^.

### Estresse oxidativo, inflamação e o rim

O estresse oxidativo (EO) ocorre quando o sistema enzimático antioxidante falha ou quando há aumento na produção de espécies reativas de oxigênio (ROS) e espécies reativas de nitrogênio (RNS)^7^. Antioxidantes endógenos, como glutationa peroxidases (GPxs), tiorredoxina (Trx), catalase (CAT) e superóxido dismutase (SOD), auxiliam na supressão do excesso de ROS e na manutenção do equilíbrio redox celular^8^. A autofagia é um processo celular vital influenciado por ROS e pelo EO. Ela envolve a degradação e a reciclagem de organelas e proteínas danificadas para manter a homeostase celular^8^. No entanto, o EO pode causar dano tecidual, inflamação, morte celular e promover a progressão de lesões e doenças^9^. Como o rim apresenta elevada taxa respiratória e grande quantidade de mitocôndrias para atender às suas altas demandas metabólicas, ele é particularmente vulnerável ao dano oxidativo e à geração de ROS^10^. Independentemente da causa, o EO, a ativação da autofagia e a disfunção mitocondrial estão inter-relacionados no desenvolvimento das doenças renais e frequentemente ocorrem à medida que as lesões renais agudas e crônicas se agravam. Diversos estudos indicam que as ROS desempenham papéis importantes no desenvolvimento da injúria renal aguda (IRA) e da doença renal crônica (DRC), tanto em contextos clínicos quanto pré-clínicos^8^. A busca contínua por novas opções terapêuticas é impulsionada pela proteção limitada oferecida pelos tratamentos tradicionais. Uma revisão sistemática de estudos clínicos sobre a eficácia e o impacto potencial de compostos bioativos naturais de plantas (LCPNs) em doenças renais constatou que vários LCPNs, incluindo o chá verde (*Camellia sinensis*), apresentam efeitos protetores, principalmente por meio da ativação de vias antioxidantes e do bloqueio de vias de sinalização pró-inflamatórias^11^.

#### Considerações de segurança e riscos potenciais associados aos chás de ervas

Embora amplamente utilizados e geralmente considerados seguros, produtos de origem vegetal, incluindo chás de ervas, apresentam certos riscos. Evidências demonstram de forma consistente que preparações herbais podem causar eventos adversos, principalmente hepatotoxicidade, particularmente quando utilizadas como suplementos alimentares ou extratos concentrados^12,13^. A lesão hepática induzida por produtos herbais é cada vez mais reconhecida na prática clínica, provavelmente devido ao aumento do consumo global desses produtos e à disponibilidade de formulações com qualidade variável^12^. Além disso, fatores como diferenças na composição química, métodos de extração e possível contaminação podem afetar significativamente sua segurança, levando a eventos adversos, sobretudo em indivíduos mais vulneráveis^13,14^. É importante destacar que os riscos são maiores com extratos concentrados, que envolvem maior exposição a compostos bioativos em doses mais elevadas e apresentam grande variabilidade entre os produtos, refletindo a falta de padronização entre muitas opções disponíveis^13,14^. Portanto, recomenda-se uma abordagem cautelosa ao consumo de chás de ervas, particularmente em populações com maior vulnerabilidade clínica, ressaltando a necessidade de avaliações individualizadas. Especificamente, no que diz respeito ao chá verde (*Camellia sinensis*), a maioria dos eventos adversos está associada a extratos concentrados ricos em catequinas, especialmente quando utilizados em altas doses ou em jejum, enquanto a infusão tradicional geralmente apresenta um perfil de segurança mais favorável, embora eventos adversos raros também tenham sido relatados^13,15^.

#### Chá verde (*Camellia sinensis*) – propriedades físico-químicas e biológicas

*Camellia sinensis* é uma espécie da família Theaceae, comumente conhecida como chá. Trata-se de uma árvore que pode atingir até 15 metros de altura, nativa das florestas do nordeste da Índia e do sul da China. O chá é a segunda bebida mais consumida no mundo, atrás apenas da água. As folhas de *Camellia sinensis* são utilizadas para sua produção. Os componentes químicos ativos da bebida têm sido amplamente caracterizados por meio de tecnologias avançadas ao longo dos últimos 40 anos^16^. Entre os aproximadamente 400 compostos químicos presentes no chá, destacam-se os compostos polifenólicos, ou flavonoides, que podem auxiliar na prevenção de doenças renais devido às suas propriedades anti-inflamatórias, antioxidantes e antiapoptóticas^11,16^.

O extrato de chá verde (*Camellia sinensis*) é obtido a partir de folhas que passam por processamento mínimo para preservar compostos fenólicos, especialmente as catequinas, que constituem um importante grupo de flavonoides^17,18^. Diferentemente do chá preto (fermentado) e do chá oolong (semi-fermentado), o chá verde é classificado como não fermentado porque as folhas são rapidamente aquecidas após a colheita, evitando a degradação das catequinas^17^. Esse processo é geralmente realizado por métodos como vaporização ou leve torrefação, que inibem a atividade da polifenol oxidase e ajudam a preservar os compostos ativos do chá^17^. Posteriormente, as folhas são cuidadosamente secas, normalmente com ar aquecido, para manter a estabilidade dos compostos bioativos^17^. O material vegetal é então moído e submetido à extração utilizando água quente ou solventes hidroalcoólicos, como etanol em diferentes concentrações, a fim de maximizar a recuperação de polifenóis, especialmente das catequinas^19^. O extrato pode ainda passar por etapas adicionais de purificação, incluindo filtração, concentração a vácuo e enriquecimento em catequinas. Por fim, o produto é seco por técnicas como *spray drying* ou liofilização, resultando em um extrato em pó padronizado, com maior teor de catequinas do que o normalmente encontrado nas infusões tradicionais de chá^15,17^. É importante destacar que diferenças nos métodos de processamento e extração podem impactar significativamente a composição final, levando a variações nos níveis de catequinas e influenciando a posologia e as vias de administração empregadas em estudos experimentais e clínicos^15,18^.

O chá verde contém cinco catequinas, das quais apenas a epicatequina-3-galato (ECG) e a epigalocatequina-3-galato (EGCG) possuem um grupo galoil. Dentre essas, a EGCG é a mais abundante, correspondendo a 50–80% do conteúdo total de catequinas. Diversos estudos demonstraram que esse grupo funcional é crucial para suas propriedades biológicas, incluindo efeitos antioxidantes^20^. No entanto, pesquisas recentes forneceram uma base teórica para o mecanismo anti-inflamatório das catequinas galoiladas (ECG e EGCG) e demonstraram que a presença do grupo galoil é essencial para suas propriedades anti-inflamatórias^21^. A EGCG é absorvida passivamente no duodeno, favorecida pelo pH alcalino do intestino. Isso ocorre porque o intestino delgado não possui um transportador ativo capaz de conduzir a EGCG para dentro dos enterócitos e, posteriormente, para a corrente sanguínea^22^. A EGCG livre apresenta biodisponibilidade oral limitada, principalmente devido à baixa absorção e à sua instabilidade intrínseca. Sua meia-vida biológica é de aproximadamente 2 a 5 horas. Portanto, cerca de 24 horas após a ingestão, os níveis sanguíneos de EGCG diminuem gradualmente até se tornarem indetectáveis^22^. Embora a EGCG seja geralmente bem tolerada, alguns estudos indicam que a ingestão diária de 800 mg ou mais pode elevar os níveis de transaminases séricas^23^. Atualmente, a EGCG está disponível apenas como suplemento alimentar, na forma de extrato de chá verde ou de produtos de natureza farmacêutica^22^. Avanços recentes no desenvolvimento de derivados de EGCG têm demonstrado propriedades promissoras, notadamente efeitos antioxidantes e anti-inflamatórios. Importante destacar que os derivados modificados mais eficazes tendem a apresentar maior estabilidade química e atividade biológica aprimorada em comparação com a EGCG não modificada. Além disso, a EGCG é menos estável em solução do que em sua forma sólida^22^. Considerando as propriedades antioxidantes das catequinas, a distribuição dos grupos hidroxila é tão importante quanto sua quantidade total. Catequinas com grupo pirogalol geralmente apresentam maior atividade antioxidante do que aquelas que possuem apenas grupos catecol. Entretanto, fatores ambientais também influenciam a eficiência antioxidante das catequinas, além de sua estrutura química^21,24^. O potencial terapêutico da EGCG tem sido amplamente investigado em diversas doenças renais relacionadas à inflamação e ao EO, em razão das atividades anti-inflamatórias, antiapoptóticas, antioxidantes e imunorregulatórias.

Assim, os potenciais benefícios do chá verde estão relacionados aos efeitos da EGCG, o polifenol mais amplamente estudado em pesquisas médicas. As evidências atuais sugerem que as propriedades desse composto podem auxiliar tanto na prevenção quanto no tratamento de doenças renais^11,16^.

#### EGCG – mecanismo de ação

O mecanismo de ação das catequinas do chá verde ainda não está completamente elucidado. Segundo diversos autores, as propriedades antioxidantes das catequinas são explicadas por uma combinação de processos diretos e indiretos. Os processos diretos envolvem a eliminação e a quelação de íons metálicos, como o ferro, que contribuem para o EO. Um exemplo de processo indireto é a regulação positiva de enzimas antioxidantes, como SOD, CAT e GPx, essenciais para a remoção de ROS. Em resposta ao EO, é importante suprimir a ativação de mediadores pró-inflamatórios e fatores de transcrição essenciais, incluindo o fator de necrose tumoral alfa (TNF-α), o fator nuclear kappa B (NF-κB) e a proteína ativadora 1 (AP-1), bem como inibir enzimas envolvidas na geração de espécies reativas de oxigênio e nitrogênio, incluindo a NADPH oxidase (NOX), a ciclooxigenase (COX), a óxido nítrico sintase induzível (iNOS), a xantina oxidase e a lipoxigenase^2^⁵ (Figura 1). Diferentes vias moleculares, principalmente aquelas envolvidas na supressão direta do EO induzido por diferentes estímulos, mediam os efeitos benéficos das catequinas do chá verde. Outros pesquisadores sustentam que as propriedades antioxidantes do chá verde decorrem da prevenção da disfunção mitocondrial e da ativação de enzimas antioxidantes, como heme oxigenase-1 (HO-1), SOD, GPx e CAT, por meio da via de sinalização Nrf2/Keap1/HO-1. Os efeitos anti-inflamatórios das catequinas ocorrem pela inibição da sinalização do receptor Toll-like 4 (TLR4) e pela supressão de NF-κB e NLRP3. Essas ações combinadas reduzem a cascata inflamatória subjacente^16^ (Figura 2). O NF-κB é considerado um dos principais reguladores pró-inflamatórios da expressão gênica, promovendo a transcrição de genes que codificam citocinas pró-inflamatórias, como TNF-α, IL-1β, IL-6 e IL-8, além de enzimas inflamatórias, incluindo a ciclooxigenase-2 (COX-2)^26^. As catequinas do chá verde apresentam efeitos anti-inflamatórios por meio de diversas vias de sinalização. Elas podem exercer tanto ações anti-inflamatórias quanto antioxidantes, sendo o EO considerado seu principal alvo molecular. Além disso, diversos pesquisadores sugerem que as catequinas do chá podem atuar como sequestradoras de ROS, que são conhecidas por ativar a via de sinalização do NF-κB, levando ao aumento da produção de citocinas pró-inflamatórias e de enzimas, como TNF-α, IL-1β, COX-2 e metaloproteinase de matriz-9 (MMP-9) (Figura 3). Portanto, as propriedades antioxidantes do chá verde podem ajudar a explicar seus efeitos anti-inflamatórios ao prevenir a ativação do NF-κB^27^.

**Figura 1:** Propriedades antioxidantes das catequinas.

**Figura 2:** Mecanismos moleculares renoprotetores da EGCG. A EGCG inibe especificamente a geração excessiva de ROS desencadeada por moléculas sinalizadoras e, indiretamente, por meio da ativação da via de sinalização Nrf2/Keap1/Cul-3, promovendo a translocação do Nrf2 livre para o núcleo, onde se liga ao ARE, ativando enzimas antioxidantes e inibindo a produção de ROS mitocondriais. A inflamação pode ser inibida pela EGCG por meio das vias de sinalização TLR4 e NF-κB/NLRP3, culminando na inibição da cascata inflamatória.

Abreviaturas – DAMPs: padrões moleculares associados a dano; TLR4: receptor Toll-like 4; STAT1: transdutor de sinal e ativador de transcrição 1; NF-κB: fator nuclear kappa B; AP-1: proteína ativadora 1; iNOS: óxido nítrico sintase induzível; COX-2: ciclooxigenase-2; TNF-α: fator de necrose tumoral alfa; IL-1β: interleucina-1β; IL-6: interleucina-6; MCP-1: proteína quimiotática de monócitos-1; SOD: superóxido dismutase; HO-1: heme oxigenase-1; GPx: glutationa peroxidase; Nrf2: fator nuclear eritroide 2 relacionado ao fator 2; ARE: elemento de resposta antioxidante; Keap1: proteína 1 associada ao tipo Kelch semelhante a ECH; Cul-3: cullin-3; E2: enzima conjugadora de ubiquitina E2; H_2_O_2_: peróxido de hidrogênio; EGCG: epigalocatequina-3-galato; 8-OHdG: 8-hidroxi-2’-desoxiguanosina; MDA: malondialdeído; MPO: mieloperoxidase; ROS: espécies reativas de oxigênio.

**Figura 3:** Atividade anti-inflamatória da EGCG. A EGCG apresenta atividade anti-inflamatória ao reduzir as ROS e suprimir a sinalização do NF-κB e seus efeitos a jusante.

Em contraste, Ayusso et al. propuseram recentemente que os efeitos anti-inflamatórios do extrato de chá verde podem resultar da inibição direta da cascata inflamatória (envolvendo TLR4, NF-κB, COX-2, IL-1β, MCP-1), juntamente com a ativação da citocina IL-6 e do eixo IL-10/HO-1 em macrófagos M2, que apresentam um fenótipo anti-inflamatório. Esse efeito pode ocorrer independentemente de sua ação antioxidante^6^ (Figura 4). Diante dessas evidências, o objetivo desta revisão foi analisar a literatura sobre os efeitos renoprotetores do chá verde (*Camellia sinensis*) em doenças renais e os mecanismos envolvidos em cada caso.

**Figura 4:** Propriedades anti-inflamatórias do extrato de chá verde. Macrófagos M1: o GTE suprime especificamente as vias de sinalização TLR4 e NF-κB, levando à supressão da cascata inflamatória subjacente. Eixo IL-10/HO-1 em macrófagos M2. A IL-10 liga-se ao seu receptor na membrana celular e promove a indução de JAK, que desencadeia o recrutamento e a subsequente ativação do STAT3 citosólico. O STAT3 é então translocado para o núcleo para aumentar a expressão de HO-1. Por fim, tanto a HO-1 quanto o CO desempenham papel na modulação da produção de IL-10 por meio da ativação da p38 MAPK.

Abreviaturas – DAMPs: padrões moleculares associados a dano; TLR4: receptor Toll-like 4; NF-κB: fator nuclear kappa B; COX-2: ciclooxigenase-2; IL-1β: interleucina-1β; MCP-1: proteína quimiotática de monócitos-1; GTE: extrato de chá verde; IL-10: interleucina-10; HO-1: heme oxigenase-1; IL-10R: receptor de interleucina-10; STAT3: transdutor de sinal e ativador de transcrição 3; SBE: elemento de ligação de STAT; CO: monóxido de carbono; JAK1/3: quinase Janus; MAPK: proteína quinase ativada por mitógeno; TYK2: tirosina quinase 2.

### MÉTODOS

Uma revisão narrativa foi realizada por meio de uma busca estruturada na base de dados PubMed/MEDLINE para identificar estudos publicados entre janeiro de 2014 e dezembro de 2024 que investigaram os efeitos do chá verde e de seu principal composto bioativo, a EGCG, em doenças renais. A busca foi conduzida em dezembro de 2024, data estabelecida como ponto de corte para a inclusão dos estudos elegíveis. Um total de 2.190 registros foi identificado, não sendo encontradas duplicatas durante o processo de seleção. Após a triagem de títulos e resumos, 2.000 artigos foram excluídos por não serem relevantes para o tema. Os 190 estudos restantes foram avaliados na íntegra, dos quais 127 foram excluídos por não preencherem os critérios de elegibilidade. Ao final, 63 estudos foram incluídos nesta revisão.

Para a identificação dos artigos, foi utilizada uma combinação de descritores controlados e termos livres relacionados ao chá verde, seus principais compostos bioativos e diversas condições renais. Foram utilizados termos como *green tea*, *Camellia sinensis*, *epigallocatechin gallate*, EGCG, *oxidative stress*, *kidney disease*, *renal disease*, *acute kidney injury*, *chronic kidney disease*, *nephrolithiasis* e *diabetic nephropathy*. Esses termos foram combinados com os operadores booleanos AND e OR para aumentar a sensibilidade da busca. Um exemplo da estratégia de busca foi: (“green tea” OR “*Camellia sinensis*” OR EGCG OR “epigallocatechin gallate”) AND (“kidney disease” OR “renal disease” OR “acute kidney injury” OR “chronic kidney disease” OR “nephrolithiasis” OR “diabetic nephropathy”) AND (“oxidative stress”).

#### Critérios de inclusão

Foram incluídos estudos originais experimentais e clínicos que investigaram os efeitos renoprotetores do chá verde ou de seu principal polifenol bioativo, a EGCG, em diferentes doenças renais. Os artigos elegíveis foram publicados entre janeiro de 2014 e dezembro de 2024, redigidos em língua inglesa e indexados na base de dados PubMed/MEDLINE.

#### Critérios de exclusão

Foram excluídos estudos que não investigaram especificamente os efeitos do chá verde ou de seus compostos bioativos em doenças renais, bem como artigos de revisão, cartas ao editor, editoriais, resumos de congressos sem acesso ao texto completo e publicações sem dados experimentais ou clínicos originais relevantes aos objetivos desta revisão.

O processo de seleção dos estudos está resumido no fluxograma (Figura S1).

## RESULTADOS

### Efeito protetor do chá verde em doenças renais

#### Doença renal crônica (DRC)

A DRC apresenta prevalência crescente em todo o mundo e está associada ao diabetes e à hipertensão. Múltiplas vias de sinalização molecular estão envolvidas na iniciação e na progressão da lesão renal. O sistema renina–angiotensina–aldosterona e a estimulação de fatores de crescimento profibróticos, como o fator de crescimento transformador β (TGF-β) e o fator de crescimento do tecido conjuntivo, fazem parte desse processo. O TGF-β aumenta a produção de ROS, suprime o sistema antioxidante e induz EO ou desequilíbrio redox celular. Essa cascata leva ao acúmulo de matriz extracelular (MEC), à fibrose renal, à disfunção epitelial e às respostas pró-inflamatórias. Essas vias não apenas se sobrepõem, mas também interagem, modificando seus papéis biológicos e promovendo a progressão da fibrose renal e da DRC^11^. Uma revisão recente da literatura concentrou-se nas principais fontes de ROS, nas defesas antioxidantes endógenas e nas vias de sinalização sensíveis ao estado redox envolvidas na regulação da inflamação e da fibrose na DRC. Os autores observaram que o sistema enzimático antioxidante (por exemplo, SOD, CAT, GPx) desempenha um papel crucial na atenuação da lesão renal induzida por ROS. Em contraste, a NADPH oxidase e as mitocôndrias são as principais fontes de ROS no rim^28^. As opções terapêuticas para prevenir a progressão dessas alterações patológicas são limitadas. Fármacos que bloqueiam o sistema renina–angiotensina–aldosterona apenas reduzem parcialmente a proteinúria, melhoram a função renal e retardam a progressão para a doença renal em estágio terminal^29^. Os efeitos protetores da EGCG no rim têm sido amplamente estudados em modelos pré-clínicos de fibrose renal e DRC.

A EGCG atua como antioxidante ao ativar a via Nrf2–HO-1, levando à produção de enzimas antioxidantes (SOD, CAT e GPx) e contribuindo para a manutenção da função mitocondrial. Seus efeitos antifibróticos são alcançados pela supressão da via TGF-β/Smad e da transição epitélio-mesênquima. Além disso, a EGCG apresenta propriedades antiapoptóticas ao inibir a sinalização da p38 MAPK. Seu efeito anti-inflamatório decorre do bloqueio da via NF-κB, que regula a expressão de quimiocinas e citocinas pró-inflamatórias, como TNF-α, IL-1β e IL-6, bem como a atividade da enzima mieloperoxidase^28,30^. Diversos estudos demonstraram que o tratamento com EGCG reduz os níveis de creatinina sérica em modelos pré-clínicos de nefropatia obstrutiva^16^.

#### Nefrolitíase

Recentemente, foi confirmado que a lesão das células epiteliais renais relacionada à formação de cálculos de oxalato de cálcio (CaOx) é mediada pelo EO, produzido pelas mitocôndrias ou pela NADPH oxidase^31^. Os papéis do EO e do estresse do retículo endoplasmático na nefrolitíase por cálcio sugerem que a conexão entre a toxicidade do oxalato e o estresse do retículo endoplasmático pode contribuir para a formação de cálculos. No entanto, a interação entre ROS e o estresse do retículo endoplasmático permanece controversa. Estudos demonstram que a formação de cristais de CaOx e o EO são fatores-chave no desenvolvimento da nefrolitíase^32^. A administração de polifenóis do chá verde inibe a produção de radicais livres induzida pelo monohidrato de oxalato de cálcio (COM) em células epiteliais tubulares renais, promove a formação de dihidrato de oxalato de cálcio (COD) e reduz a expressão de α-enolase (um receptor para COM cristalino) na superfície celular, além de diminuir a oxalúria. A forma dos cristais e dos agregados celulares renais pode influenciar a adesão do CaOx às células tubulares renais, processo crítico para o desenvolvimento dos cálculos. Uma via de sinalização desencadeada pelas catequinas do chá verde que combate o EO relacionado à formação de cálculos renais envolve o aumento dos níveis de enzimas antioxidantes, como SOD, GPx e CAT^33,34^.

Poucos ensaios clínicos exploraram a relação entre o chá verde e a nefrolitíase. Um grande estudo de coorte realizado no Reino Unido, envolvendo 439.072 participantes acompanhados por uma média superior a seis anos, constatou que indivíduos que consumiam cinco xícaras de chá verde por dia apresentavam risco significativamente menor de cálculos renais em comparação àqueles que não consumiam chá regularmente^34^. Recentemente, uma revisão sistemática avaliou as evidências disponíveis sobre a relação entre o consumo de chá e o risco de nefrolitíase^35^. A revisão incluiu 13 estudos não randomizados, com desenhos variados, e carecia de métodos padronizados de preparo do chá, indicando viés de publicação. Devido às diferenças nos procedimentos e nos dados, não foi possível realizar uma metanálise. A revisão sugeriu que o chá poderia auxiliar na prevenção de cálculos renais devido aos seus efeitos antioxidantes, benefícios de hidratação e teor de cafeína^35^.

Um estudo de coorte prospectivo recente, realizado na China e envolvendo 502.621 adultos acompanhados por uma mediana superior a 11 anos, demonstrou que o consumo de três xícaras de chá por dia, ingestão de 30 g de álcool e consumo de frutas quatro vezes por semana estavam associados a menor incidência de cálculos renais^36^. Esses fatores foram independentemente associados a menores taxas de nefrolitíase^36^. Fatores nutricionais influenciam significativamente o desenvolvimento da urolitíase. Uma revisão narrativa forneceu uma visão abrangente e atualizada dos efeitos da nutrição e da dieta sobre a nefrolitíase. Estudos de coorte de grande porte que sustentam os benefícios do consumo de café e chá sugerem que os efeitos positivos do chá se devem principalmente às suas propriedades diuréticas, relacionadas à cafeína, que podem contribuir para a redução da hipercalciúria. As propriedades antioxidantes dos polifenóis também podem ajudar a explicar os efeitos protetores do chá. No entanto, esses estudos de coorte apresentaram uma limitação: não diferenciaram entre chá preto e chá verde, que possuem níveis distintos de oxalato, dependendo de fatores como origem, qualidade, época de colheita e métodos de preparo. Portanto, os mecanismos exatos pelos quais o chá preto e o chá verde inibem a formação de cálculos permanecem não totalmente elucidados^37^.

#### Nefropatia diabética

O diabetes mellitus pode levar a complicações macrovasculares e microvasculares, incluindo a nefropatia diabética (ND), uma doença microvascular amplamente temida na prática clínica. A ND envolve comprometimento da filtração renal e desenvolvimento de proteinúria. Estudos experimentais e em humanos demonstraram que o chá exerce efeitos protetores contra o diabetes mellitus e auxilia no manejo de suas complicações associadas. Evidências sugerem que o chá pode influenciar os mecanismos subjacentes à ND por meio de suas propriedades antioxidantes, anti-inflamatórias^38,39^, antiapoptóticas e antifibróticas^16,40^, reduzindo, assim, a proteinúria e preservando a função renal^16,38,40^. Um ensaio clínico randomizado, duplo-cego e controlado por placebo, envolvendo 42 pacientes com ND, demonstrou que o extrato de chá verde (polifenóis), quando utilizado em associação com doses máximas de bloqueadores do receptor de angiotensina II (BRAs) e/ou inibidores da enzima conversora de angiotensina (IECA), foi seguro e eficaz, resultando em redução da proteinúria e em menor apoptose de podócitos por meio da via Wnt^41^.

Estudos epidemiológicos multinacionais realizados nos Estados Unidos, China, Japão e Coreia do Sul, envolvendo populações com diversidade de raça, sexo e faixa etária, demonstraram que o consumo de chá pode reduzir o risco de desenvolvimento de diabetes e de suas complicações, incluindo a nefropatia. A maioria dos participantes consumia chá preto, chá oolong ou chá verde. O antioxidante EGCG pode reduzir o EO e a inflamação por meio da indução da via de sinalização Nrf2 e da inibição da via NF-κB, respectivamente. Devido à variabilidade das doses de chá avaliadas e aos efeitos observados em estudos pré-clínicos e clínicos, ainda há incerteza quanto à capacidade de doses eficazes em modelos pré-clínicos produzirem benefícios semelhantes em humanos^38^.

A hiperglicemia pode desencadear EO, formação de produtos finais de glicação avançada (AGEs), ativação da via do poliol e ativação anormal da proteína quinase C (PKC), processos que contribuem para o desenvolvimento da ND. Níveis elevados de glicose sanguínea aumentam a síntese de α-diacilglicerol (α-DAG), o que ativa de forma desregulada a PKC, levando à ND e a outras complicações vasculares. A regulação positiva da α-diacilglicerol quinase (α-DGK) inibe a ativação da PKC e reduz os níveis de α-DAG. Estudos pré-clínicos em camundongos diabéticos tipo I induzidos por estreptozotocina demonstraram que a EGCG reduz significativamente a albuminúria na ND por meio da ativação da via de sinalização α-DGK. Esses estudos também evidenciaram que a EGCG ativa a α-DGK por meio do receptor de laminina de 67 kDa (67LR), conhecido como receptor da EGCG. Os achados sugerem que a EGCG desencadeia a ativação da α-DGK por meio do 67LR e da c-Src, com a α-DGK associando-se ao 67LR nas membranas dos podócitos para inibir a PKC. Após a ativação induzida pela EGCG, complexos de α-DGK, c-Src e 67LR parecem ser formados. Os pesquisadores concluíram que a ativação da via α-DGK pode representar um novo alvo terapêutico promissor para a ND^42^.

Estratégias para retardar a progressão da ND incluem modificações no estilo de vida, orientação dietética, prática regular de atividade física e o uso de fármacos renoprotetores, como BRAs e IECAs. A principal via bioquímica envolvida na progressão da doença é mediada pelos AGEs, que se ligam ao seu receptor (RAGE) e modulam a sinalização intracelular, podendo promover ou agravar a lesão renal. O RAGE solúvel (sRAGE), uma variante do RAGE, interage com os AGEs e pode contribuir para a inibição dessa via de sinalização. Um dos mecanismos propostos para o desenvolvimento da ND envolve níveis elevados de AGEs que se ligam ao RAGE, causando danos às células do néfron. O chá verde tem demonstrado potencial para aumentar a expressão de sRAGE; entretanto, permanece incerto se esse efeito resulta em melhora da função renal. Um ensaio clínico randomizado, duplo-cego e controlado por placebo, conduzido em Guadalajara, no México, avaliou o impacto do extrato de chá verde nos níveis de sRAGE e no dano renal em indivíduos com diabetes tipo 2^43^. O estudo incluiu 39 homens e mulheres com idades entre 35 e 65 anos, todos em uso de hipoglicemiantes orais e/ou insulina, com DRC estágios 2 ou 3a de acordo com as diretrizes KDIGO, e níveis de HbA1c entre 9% e 12%. Os participantes receberam 400 mg de chá verde duas vezes ao dia ou placebo por três meses. Os pesquisadores observaram que o extrato de chá verde aumentou significativamente os níveis séricos de sRAGE e a taxa de filtração glomerular, além de reduzir a glicemia de jejum e os níveis de triglicerídeos^43^.

#### Injúria renal aguda

A injúria renal aguda (IRA) refere-se a uma perda súbita da função renal. A creatinina sérica e o débito urinário são indicadores-chave para o diagnóstico de IRA, embora suas limitações sejam amplamente reconhecidas^44^. A IRA permanece um importante problema de saúde global, afetando aproximadamente 13,3 milhões de pessoas em todo o mundo a cada ano^45^. Durante a pandemia de COVID-19, a IRA foi frequente e associada a maiores taxas de morbidade e mortalidade. Diversos estudos demonstraram que a EGCG pode atuar como agente protetor ou terapêutico na IRA. Seus efeitos protetores podem incluir atividades antioxidantes, anti-inflamatórias e antiapoptóticas. As principais vias de sinalização envolvidas são a ativação da via Nrf2–HO-1 e a supressão do NF-κB por meio da MAPK^16,46^. Estudos em ratos pré-tratados ou tratados com catequinas demonstraram proteção significativa ou redução do dano renal, incluindo lesões glomerulares e tubulares, em modelos de IRA induzida por gentamicina, contraste iodado, rabdomiólise, cisplatina, glicerol, radiação gama, estresse térmico, obstrução, isquemia/reperfusão, circulação extracorpórea, sobrecarga de ferro, ciclosporina, tacrolimo e doxorrubicina^6,16,42,47,48,49,50,51,52,53^. Pesquisas recentes indicam que as catequinas também apresentam efeitos antivirais e podem prevenir a IRA associada à COVID-19^45,54^. As catequinas demonstraram propriedades antivirais contra coronavírus e outros vírus de RNA, podendo inibir endocitose, replicação e transcrição por mecanismos que ainda não estão completamente elucidados. Está bem estabelecido que a EGCG interage com o receptor ACE2 presente nas membranas das células hospedeiras, bem como com a RNA polimerase dependente de RNA (RdRp) e a protease principal (Mpro), inibindo, assim, a replicação do SARS-CoV-2. O receptor ACE2 atua como sítio de ligação para a proteína *spike* do SARS-CoV-2, especificamente por meio do domínio de ligação ao receptor (RBD) presente na subunidade S1 viral^54^. A interação entre a proteína *spike* do SARS-CoV-2 e o receptor ACE2 é mediada pelo RBD, localizado na subunidade S1. Essa interação facilita o reconhecimento do ACE2 pelo RBD e a formação do complexo RBD–ACE2. Uma vez formado esse complexo, o SARS-CoV-2 torna-se apto à endocitose, iniciando o processo de replicação. Em estudos *in vitro*, demonstrou-se que a EGCG se liga à proteína *spike* do SARS-CoV-2, bloqueando sua interação com o ACE2 nas células hospedeiras e impedindo a entrada viral^45^. Além disso, a EGCG inibe etapas iniciais da replicação viral, tendo como alvo a Mpro e a RdRp^55^. Esses achados sustentam o potencial das catequinas do chá como estratégia terapêutica renoprotetora na IRA associada à COVID-19, devido às suas propriedades anti-SARS-CoV-2, antioxidantes e anti-inflamatórias^45^.

A Tabela 1 resume os principais estudos experimentais e clínicos incluídos nesta revisão, destacando as vias de administração, as doses empregadas, a duração das intervenções, os principais desfechos renais avaliados e o perfil de segurança do chá verde e de suas catequinas, especialmente a EGCG.

**Tabela 1:** Síntese dos principais estudos sobre chá verde e EGCG em doenças renais.

Estudo (autor, ano)	Condição	Desenho do estudo	Administração	Dose	Duração	Desfecho principal	Segurança
Li et al., 2021	Nefrolitíase	Experimental	Polifenóis (gavagem)	30–300 mg/kg/dia	4 semanas	↓ formação de cristais	Bem tolerado
Littlejohns et al., 2019	Nefrolitíase	Coorte	Infusão de chá	~5 xícaras/dia	> 6 anos	↓ risco de cálculos	Sem efeitos adversos significativos
Wang et al., 2021	Nefrolitíase	Coorte	Infusão de chá	≥ 3 xícaras/dia	> 11 anos	↓ incidência de nefrolitíase	Sem efeitos adversos significativos
Borges et al., 2016	Nefropatia diabética	Ensaio clínico randomizado (ECR)	Extrato	400 mg, duas vezes ao dia	12 semanas	↓ proteinúria	Bem tolerado
Hayashi et al., 2020	Nefropatia diabética	Experimental	EGCG (modelo com STZ)	0,1% na dieta (w/w)	8 semanas	↓ albuminúria	Bem tolerado
Barocio et al., 2021	Nefropatia diabética	Ensaio clínico randomizado (ECR)	Extrato	400 mg, duas vezes ao dia	3 meses	↑ função renal e parâmetros metabólicos	Bem tolerado
Múltiplos estudos	IRA	Experimental	EGCG/extrato	Variável	Variável	Proteção tubular	Potencial hepatotoxicidade em doses elevadas

## DISCUSSÃO E CONCLUSÕES

Medicamentos fitoterápicos frequentemente enfrentam desafios, incluindo falta de padronização, variabilidade na composição e controle de qualidade inadequado, o que pode impactar sua segurança e confiabilidade clínica. Esses problemas são agravados por diferenças nos marcos regulatórios e nos sistemas de farmacovigilância entre países, especialmente em regiões de baixa e média renda, incluindo o Brasil^56^. A segurança das catequinas do chá verde, especialmente da EGCG, deve ser considerada ao avaliar seu potencial terapêutico em doenças renais. Evidências da *European Food Safety Authority* demonstram que doses elevadas, particularmente ≥ 800 mg/dia de extratos concentrados, podem causar alterações hepáticas^15,23^, enquanto doses mais baixas não têm sido consistentemente associadas à toxicidade hepática. Fatores como administração em bolus, estado de jejum e características da formulação também podem influenciar seu perfil de segurança^13,15^. Ainda assim, dados da *United States Pharmacopeia* sugerem que a hepatotoxicidade grave associada a extratos de chá verde é rara, apesar de seu amplo uso mundial^13^. Quando eventos adversos ocorrem, eles parecem estar principalmente relacionados a fatores individuais, incluindo predisposição genética, reações idiossincráticas, uso de fármacos hepatotóxicos, doença hepática pré-existente e condições de formulação e uso^13,15^. Estudos clínicos demonstram que a EGCG apresenta boa tolerabilidade, especialmente em doses moderadas^41,43^, sem sinais consistentes de toxicidade, embora a segurança possa depender da dose e da formulação^15^. De forma consistente, ensaios clínicos conduzidos por Borges et al.^41^ e Barocio-Pantoja et al.^43^, utilizando doses de 800 mg/dia, não relataram efeitos adversos, corroborando o perfil favorável de segurança e tolerabilidade do composto (Tabela 1). O manejo da DRC, especialmente da doença renal diabética, avançou com a inclusão de terapias renoprotetoras que retardam a progressão da doença, reduzem o risco cardiovascular e diminuem a mortalidade. Entre elas destacam-se os inibidores de SGLT2 (dapagliflozina e empagliflozina), os antagonistas do receptor de mineralocorticoide (finerenona) e os agonistas do receptor de GLP-1 (liraglutida)^57,58,59,60,61^. No entanto, persiste um risco residual significativo de progressão da doença, apesar do bloqueio do sistema renina–angiotensina^62^. Diante do crescente ônus global das doenças renais^63^, novas abordagens terapêuticas ainda são necessárias. A síntese dos estudos incluídos (Figura 5 e Tabela 1) indica efeitos renoprotetores promissores da EGCG; contudo, a significativa heterogeneidade metodológica e a variabilidade nas formulações limitam sua aplicação clínica. Esses efeitos parecem envolver a modulação do EO e da inflamação, especialmente por meio das vias Nrf2–Keap1 e NF-κB. Concluímos que a EGCG pode representar um adjuvante útil às terapias atualmente estabelecidas; entretanto, ensaios clínicos bem delineados são necessários para definir seu papel na prática clínica.

**Figura 5:** Efeitos renoprotetores da EGCG em diferentes doenças renais. A EGCG pode proteger contra doenças renais por meio de diversas vias, principalmente por suas atividades antioxidantes e anti-inflamatórias.

Abreviaturas – IRA: injúria renal aguda; ND: nefropatia diabética; DRC: doença renal crônica; KS: cálculos renais; Nrf2: fator nuclear eritroide 2 relacionado ao fator 2; HO-1: heme oxigenase-1; EGCG: epigalocatequina-3-galato; NF-κB: fator nuclear kappa-light-chain-enhancer de células B ativadas; MAPK: proteína quinase ativada por mitógeno; TFG: taxa de filtração glomerular; TGF-β: fator de crescimento transformador-β; EMT: transição epitélio–mesênquima; α-DGK: α-diacilglicerol quinase; COM/COD: transformação de cristais de oxalato de cálcio monohidratado (COM) em cristais de oxalato de cálcio di-hidratado (COD).

## Data Availability

Os conjuntos de dados gerados e/ou analisados durante este estudo estão disponíveis junto ao autor correspondente, mediante solicitação razoável.

## MATERIAL SUPLEMENTAR

O seguinte material online está disponível para o presente artigo:

Figura S1 – Fluxograma de seleção dos estudos.
